# From a Hospital for the Insane: The Few November Flowers

**Published:** 1886-10-30

**Authors:** 


### THE FEW NOVEMBER FLOWERS.*

When Autumn's sere decaying leaf has all but passed away,
When th' ungenial sun appears reluctantly to stay,
The leaden skies with wintry gloom hang heavy o'er the lea
(Except when east or northern blast drives tempest to the sea),
Then most we prize, in such a dearth, those evergreens so tall,
And the simple cottage roses that adorn the southern wall.

When childhood's days of innocence and youthful glee are gone,
When care creeps in, and steals away our pleasures one by one,
The friends who once lov'd constantly are changed, or gone before,
And few are left to sympathise or flatter any more,
Then first we learn to look for peace and happiness within,
Nor trust to honour, wealth, nor power the golden joy to win!

These homely flowers and evergreens are rendered dearer far
Than when worsted and outrivalled in creation's summer war;
They remind us of resources which a pious heart can show,
Unfriended, save by welcome thought, detach'd from all below;
They are like the quiet consciousness of rectitude and peace,
They are sent as cheering foretastes of the springs that never cease!

In visiting the hospitals, where lie in pain the weak,
Restless by day—awake by night—sad pallor on the cheek—
Oh, Christian, kindly visitor, take *Flowers* in your hand,
And place a cup of water near, where they are seen to stand,
Reminding country patients of the happy days of yore,
Of lanes, and downs, and gardens, where the like once grew before;
You will cheer the suffering creatures, and make them hope again
For brighter hours to come, and a riddance of their pain.

M.A. of Christ Church, Oxford.

* Written expressly for The Hospital, by an inmate of a Hospital for the Insane, who, however, indignantly denies that his mind is affected. If to write pleasing verses is a proof of sanity, his contention can hardly be gainsaid. We hope that the poet will favour us with more "copy."—Ed. T. H.

